# Respiratory Syncytial Virus F Subunit Vaccine With AS02 Adjuvant Elicits Balanced, Robust Humoral and Cellular Immunity in BALB/c Mice

**DOI:** 10.3389/fimmu.2020.526965

**Published:** 2020-09-11

**Authors:** Yu Zheng, Lijun Bian, Huiting Zhao, Yulan Liu, Jingcai Lu, Dawei Liu, Ke Zhang, Yueshuang Song, Yusi Luo, Chunlai Jiang, Yan Chen, Yong Zhang, Wei Kong

**Affiliations:** ^1^National Engineering Laboratory for AIDS Vaccine, School of Life Sciences, Jilin University, Changchun, China; ^2^R&D Center, Changchun BCHT Biotechnology Co., Changchun, China; ^3^The Key and Characteristic Laboratory of Modern Pathogen Biology, Department of Parasitology, Basic Medical College, Guizhou Medical University, Guiyang, China; ^4^Intensive Care Unit, Department of Emergency, Guizhou Medical University Affiliated Hospital, Guiyang, China; ^5^Key Laboratory for Molecular Enzymology and Engineering, The Ministry of Education, School of Life Sciences, Jilin University, Changchun, China

**Keywords:** respiratory syncytial virus, subunit vaccine, vaccine adjuvant, humoral immunity, cellular immunity

## Abstract

Respiratory syncytial virus (RSV) is a leading cause of lower respiratory illness, particularly in infants, the elderly, and immunocompromised adults. There is no licensed commercial vaccine against RSV. Importantly, formalin-inactivated RSV vaccines mediate enhanced respiratory disease. RSV fusion (F) protein with pre-fusion conformation is a promising candidate subunit vaccine. However, some problems remain to be solved, such as low immunogenicity and humoral immunity bias. Adjuvants can effectively enhance and adjust vaccine immune responses. In this study, we formulated pre-fusion RSV-F protein with the adjuvants, Alhydrogel, MF59, AS03, AS02, and glycol chitosan (GCS). We then conducted head-to-head comparisons of vaccine-induced immune responses in BALB/c mice. All adjuvanted vaccines enhanced antigen-specific and neutralizing antibody titers and viral clearance and gave an order of adjuvant activity: AS02 > AS03, MF59 > GCS, and Alhydrogel. Among them, AS02 elicited the highest antibody expression, which persisted until week 18. Moreover, AS02 significantly enhanced Th1 type immune response in immunized mice. Mice in the AS02 group also showed faster recovery from viral attacks in challenge tests. Further transcriptome analysis revealed that AS02 regulates immune balance by activating TLR-4 and promotes Th1-type immune responses. These results suggest that AS02 may be an excellent candidate adjuvant for RSV-F subunit vaccines. This study also provides valuable information regarding the effect of other adjuvants on immune responses of RSV-F subunit vaccines.

## Introduction

Respiratory syncytial virus (RSV) is a leading cause of lower respiratory illness, particularly in infants, the elderly, and immunocompromised adults. Currently, there is no licensed commercial vaccine to treat RSV (1, 2). The development of RSV vaccines began in 1960, when the formalin-inactivated RSV vaccine-mediated enhanced respiratory disease (ERD) accident caused two infant deaths. ERD is caused by a strong T-helper 2 (Th2) cell-biased immune response and antibodies with low functional activity (3). Targeted subunit vaccine construction can improve vaccine specificity and safety (4, 5). There are many target candidates for developing RSV subunit vaccines. Among the eleven RSV-encoded proteins, fusion (F) protein has viral-host membrane fusion function. F protein-targeted passive immunotherapy is safe and effective (6–8). The RSV F protein is a type I membrane protein initially synthesized as an inactive precursor protein (F0) that assembles into trimers (9). After fusion with the host cell membrane, the F protein forms a stable post-fusion conformation. Previous reports indicate that pre-fusion conformation F protein is more suitable as an antigen than post-fusion conformation F protein, because it greatly induces neutralizing antibodies (5, 10, 11). In addition, most F proteins on formalin-inactivated RSV have post-fusion conformations (12). A stable RSV-F protein with pre-fusion conformation can be achieved either by mutating the arginine residues in the two multibasic furin cleavage sites to lysines (13), or by introducing cysteine residues and filling hydrophobic cavities (14). The latter has confirmed immune efficacy in mice and macaques (13).

Respiratory syncytial virus F protein, like other subunit vaccines, usually needs to be formulated with adjuvant, due to low immunogenicity (15, 16). Combining a protein antigen with various adjuvants is a common strategy in clinical trials (8, 17). Many studies confirm that the appropriate adjuvant can significantly improve antigen-specific antibody production and even achieve a desired immune response type (18–20). For example, aluminum salts are the most common adjuvant, and can enhance Th2-biased immune responses by adsorbing antigen, causing inflammation, and promoting antigen-producing cell (APC) capture as an antigen depot (21, 22). Squalene-based oil-in-water nanoemulsion MF59 and AS03 show immune-enhancing properties, and are successfully used in influenza vaccines (23–26). AS02 is the combination of AS03 with 3-deacylated monophosphoryl lipid (MPL) and *Quillaja saponaria* fraction 21 (QS-21), which contributes to humoral and cellular immune responses (27, 28). MPL is a detoxified lipopolysaccharide (LPS) derivative consisting of a disaccharide core conjugated with varied medium-chain fatty acids (29). Additionally MPL is a TLR-4 receptor agonist, which stimulates helper T cells to produce interferon-γ (IFN-γ) and induces plasma cells to express IgG2a antibodies (30). QS-21, a saponin extracted from *Q. saponaria* tree bark, promotes antigen-specific antibody responses and CD8+ T-cell responses in mice (31). Besides squalene-based adjuvants, chitosan has also attracted attention as a vaccine adjuvant. Previous studies confirmed that chitosan promotes cellular immunity via cGAS-STING-dependent type I interferon induction and can be used as mucosal and systematic adjuvants (32–34).

In this study, we formulated pre-fusion RSV-F protein with the adjuvants Alhydrogel, MF59, AS03, AS02, and glycol chitosan (GCS). We then compared adjuvant effect on immune responses, specifically assaying antigen-specific antibodies, antibody subtype, and neutralizing antibodies in BALB/c mice. Cytokine production and the blood transcriptome were also studied to illustrate the underlying mechanisms induced by the adjuvant vaccines. Moreover, virus challenge tests were performed to assess the protective effect of different adjuvant-assisted vaccines. This study provides valuable information regarding the development of adjuvant-assisted RSV-F subunit vaccines.

## Materials and Methods

### Vaccines and Viruses

His-tag-conjugated RSV-F protein was produced and purified, based on the RSV serotype A F protein (aa 26-515) isolated from Europe (Genbank accession number JX015498.1), as previously described (13, 35). To keep RSV-F in a pre-fusion state, the arginine residues in the two multibasic furin cleavage sites were mutated to lysine residues. Briefly, the F protein-encoding sequences were cloned in-frame downstream of the pCD5 expression vector CD5 signal peptide-coding DNA sequence. The upstream sequences encode an artificial GCN4 isoleucine zipper trimerization motif and a tag which ensures the trimer structure of the expressed RSV-F protein (36, 37). The resultant pCD5 expression vectors were transfected into HEK293T (NIH) cells. Cell supernatants were harvested 5–6 days post-transfection. F protein was purified using Strep-tactin Sepharose beads (IBA, GER). The schematic representation of the recombinant soluble RSV F protein construct is showed in Supplementary Figure S1. The results of SDS-PAGE analysis and size exclusion chromatogram of F protein are shown in Supplementary Figures S2, S3, respectively. For more detailed information, please refer to previous published reports (13, 37).

Respiratory syncytial virus/A/Long strains were grown in Hep-2 (ATCC, CCL-23) cells and harvested in serum-free media by freeze-thawing twice and centrifuging at 8000 rpm for 10 min at 4°C. The virus titer was determined by plaque assays before virus challenge studies.

### Adjuvant

Monophosphoryl lipid (synthetic, molecular weight 1762.311) was purchased from Avanti Polar Lipids (Alabaster, AL, United States). QS-21 was purchased from Desert King International (San Diego, CA, United States). Adjuvants used in this study were Alhydrogel (Brenntag Biosector, Denmark), MF59, AS03, AS02, and GCS (Sigma-Aldrich, United States). MF59, AS03, and AS02 are squalene-based oil-in-water emulsions with 150–160 nm particles. Squalene-based adjuvants were prepared as previously described, and all polydispersity indexes of these adjuvants are less than 0.15 (38, 39). To ensure consistent adjuvant dose, each mouse was given an adjuvant totally containing 2.15 mg squalene at immunization. One standard dose of AS02 per mouse also contained 20 μg MPL and 15 μg QS21. The Alhydrogel and GCS doses were 100 μg and 1 mg per mouse, respectively.

### Animal Studies

**FIGURE 1:**
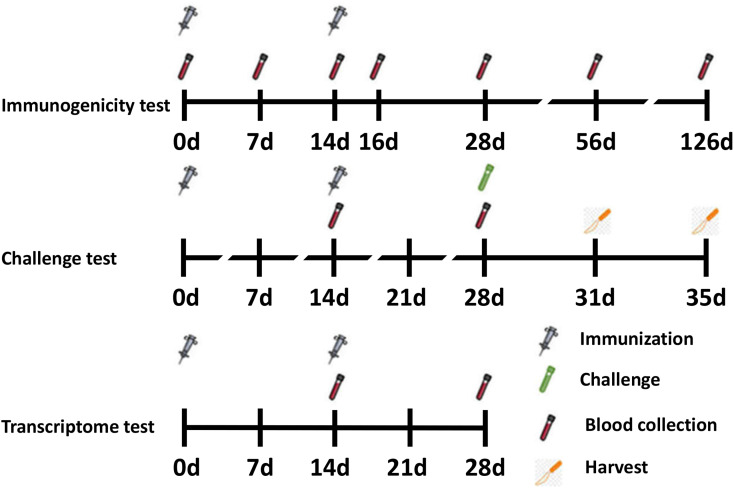
Timeline for vaccination, challenge, blood, and tissue sampling schedules.

Female BALB/c mice (6–8 weeks old) were purchased from Changchun Institute of Biological Products and randomized into seven groups (*n* = 6 per group). On days 0 and 14, 50 μL vaccines were administered intramuscularly into the thigh muscle. Vaccines consisted of 1 μg RSV-F and adjuvants grouped as follows: (1) PBS (negative control), (2) RSV-F, (3) RSV-F + Alhydrogel, (4) RSV-F + MF59, (5) RSV-F + AS02, (6) RSV-F + AS03, and (7) RSV-F + GCS. Blood samples were collected on days 7, 14, 16, 28, 56, and 126 after the first immunization (Figure 1). Samples were centrifuged twice at 3000 rpm for 8 min. Serum was collected and stored at −20°C until antibody testing.

For challenge tests, 12 mice from each group were immunized as described above and intranasally challenged with 100 μL RSV suspension (5 × 10^6^ TCID_50_ RSV/A/long per animal) 14 days after the last vaccination. Virus challenge studies were performed under pentobarbital anesthesia (50 mg/kg, intraperitoneal injection). Animals were sacrificed by cervical dislocation 3 or 5 days after challenge and the lungs were removed (Figure 1). The right lung was perfused with 4% paraformaldehyde for histopathology tests, while the lingular lobe of the left lung was homogenized and used for virus titration.

For transcriptome tests (assay details described below), mice were divided into four groups: (1) RSV-F, (2) RSV-F + Alhydrogel, (3) RSV-F + AS02, and (4) RSV-F + AS03. Mice were inoculated intramuscularly into the thigh muscle on days 0 and 14 as described above. Then, blood samples were collected on day 28 for transcriptome sequencing experiments (Figure 1) (13, 40). The sampling time point with peak immune responses was chosen to show more obvious differences between groups.

All mice used in this study were treated according to the Guide for the Care and Use of Laboratory Animals (National Research Council). And all experimental procedures were reviewed and approved by the Animal Welfare and Research Ethics Committee at Jilin University.

### Serum IgG, IgG1, and IgG2a Assay

Antibody titers were detected using enzyme-linked immunosorbent assay (ELISA). First, 96-well plates were coated with 0.25 μg RSV-F protein per well overnight at 4°C. Then, plates were blocked with 1% bovine serum albumin dissolved in PBS for 2 h at 37°C. After washing with PBS containing 0.05% Tween-80, threefold serially diluted sera were added to the wells for 1 h at 37°C. Then, anti-mouse HRP-IgG was added to detect antigen-specific antibody levels in serum samples, while goat anti-mouse IgG1 and IgG2a antibody (Sigma-Aldrich, United States, dilution ratios 1:5000) and anti-goat HRP-IgG (Jackson ImmunoResearch, United States, dilution ratio 1:10,000) were used to determine IgG1 and IgG2a antibody levels. These antibodies were incubated at 37°C for 1 h. Plates were then incubated at room temperature with 3,3′,5,5′-tetramethylbenzidine (TMB) substrate 100 μL per well in the dark for 20 min. The reaction was stopped by 2M H_2_SO_4_ after 20 min. The optical density (OD) was observed at 450 nm. Antibody titers were determined by the absorbance of a dilution ratio that was twofold greater than the average OD of the blank group. The assay limit of detection (LOD) = 100. Any sample resulting in a titer less than the LOD was assigned a value of 33.3. The geometric mean from each group was calculated ± SD.

### Serum Neutralization Assay

Serum was analyzed for neutralizing antibodies against RSV/A/Long virus in HEp-2 cells. Serum was twofold serially diluted with free medium in 96 well plates (50 μL per well). An equal volume (50 μL) of virus (10^4^ TCID_50_/mL) was added to the diluted serum, and the mixture was incubated for 1 h at 37°C. Then, 100 μL trypsinized HEp-2 cells (5 × 10^5^ cells/mL) in growth medium was added to the virus/serum mixture and incubated for 6–7 days at 37°C or until positive control (virus only) wells showed 80% cytopathic effect (CPE). Cells were fixed and stained with 0.25% crystal violet in 4% paraformaldehyde. Stained plates were air-dried and evaluated for CPE using a dissecting microscope. The lowest dilution that resulted in 80% CPE inhibition was identified as the endpoint neutralizing antibody titer for that sample. LOD was assigned as 4. Any sample with a titer less than the LOD was assigned a value of 2. Data are represented as geometric mean ± SD.

### Cytokine Measurements

Serum was collected at day 16 (2 days after the second immunization). Cytokines in the serum were assayed using a Luminex instrument and a mouse cytokine multiplex kit (LXSAHSM-07, R&D, Minnesota, MN, United States). The cytokine panel included IFN-γ, TNF, interleukin-2 (IL-2), IL-4, IL-6, IL-10, and IL-17/17A. Cytokine concentrations are expressed in pg/mL.

### Lung Viral Titers

Lungs were removed at day 3 and 5 after RSV challenge. The RSV titer was determined as described previously (41). Briefly, lungs were collected in serum-free RPMI 1640 media, weighed, and homogenized using a burnisher. The homogenates were separated by centrifuging at 3000 rpm for 10 min at room temperature. The supernatants were collected and used as the samples for titer tests. Samples were twofold serially diluted with serum-free Dulbecco’s Modified Eagle Medium (DMEM) and incubated with Hep-2 cells for 2 h at 37°C. Then, DMEM media with 10% serum and 1% agarose was added. After 6–7 days of incubation, cells in 96-well plates were fixed with 4% paraformaldehyde containing 2% crystal violet. Wells with detached cells caused by CPE were considered positive. The results are expressed as TCID_50_/0.1 g lung tissue.

### Histopathology

Samples were treated with 4% paraformaldehyde and embedded into paraffin blocks. Hematoxylin-eosin staining (HE), periodic acid schiff (PAS) staining, and pathological description were performed by a pathology sample preparation company. Histopathology staff was blinded to the experimental design. The histopathology evaluation scheme was based on Weiss et al. (42), including perivascular leukocyte aggregates (1, normal aggregation level; 2, small numbers of solitary cells with few aggregates; 3, multifocal small-to-moderate aggregates; and 4, moderate-to-high cellularity with multifocal, large cellular aggregates that may expand into adjacent tissues), interstitial disease (1, within normal parameters; 2, mild, detectable focal-to-multifocal congestion, with rare-to-few leukocytes and some atelectasis; 3, moderate, multifocal-to-coalescing congestion, leukocyte cellularity, and atelectasis, with rare luminal leakage of cellular and fluid debris; and 4, severe, coalescing interstitial congestion, leukocytes, and atelectasis, with extensive airspace loss and luminal accumulation of cellular and fluid debris), and mucus (1, none; 2, epithelial mucinous hyperplasia with none-to-rare luminal mucus; 3, epithelial mucinous hyperplasia with luminal mucus accumulation in airways; and 4, severe mucinous change with some airways completely obstructed by mucus). The sum of pathological scores were then used to evaluate the severity of each group.

### RNA Extraction and Transcriptome Sequencing

Four groups [(1) RSV-F, (2) RSV-F + Alhydrogel, (3) RSV-F + AS02, and (4) RSV-F + AS03] were analyzed in the transcriptome sequencing experiment. Fourteen days after the last vaccination, blood samples from three mice in each group were collected. Total RNA was isolated with TRIzol (Invitrogen, United States). Transcriptome sequencing and differential gene expression analysis were performed as previously described (38). RNA degradation and contamination were assayed on 1% agarose gels, and RNA purity was verified using a NanoPhotometer spectrophotometer (IMPLEN, US). Concentration were measured using a Qubit RNA Assay Kit and a Qubit 2.0 Fluorometer (Life Technologies, United States). RNA integrity was assessed using an RNA Nano 6000 Assay Kit and a Bioanalyzer 2100 system (Agilent Technologies, Untied States). Three microgram RNA per qualified sample was used as input material for RNA library preparation. Sequencing libraries were generated using a NEBNext Ultra RNA Library Prep Kit for Illumina (New England Biolabs, United States), and unique index codes were added to each sample.

### Gene Clustering and Differential Expression Analysis

Index-coded sample clustering was performed on a cBot Cluster Generation System using a TruSeq PE Cluster Kit v3-cBot-HS (Illumina, United States). Samples were sequenced on an Illumina Hiseq platform. Fragments per kilobase million (FPKM) values were calculated for each sample. Differential expression analysis was performed using the DESeq2 R package (1.10.1). Due to the large gene expression differences between samples in the same group, *P*-values were used as the standard in this experiment. Genes with *P* < 0.05 were considered differentially expressed. To characterize the differential biological function between the adjuvant group and the simple antigen group, differentially expressed genes (DEGs) were analyzed by GO enrichment analysis using the clusterProfiler R package. Gene length bias was corrected for these analyses. GO terms with *P* < 0.05 were considered significantly enriched. To further explore high-level functions and biological utility, KEGG pathway analysis was performed using the DAVID functional enrichment database referencing the public KEGG database^1^. The clusterProfiler R package was used to test DEG enrichment in KEGG pathways.

### RT-qPCR Validation

To validate the transcriptome data, Quantitative real-time polymerase chain reaction (RT-qPCR) was conducted by selecting two DEGs from each paired group (Al vs. RSV-F: P21 and Irf9, AS02 vs. RSV-F: Irf7 and Akt2, AS03 vs. RSV-F: CD21 and Gsk3a). Table 1 shows the sequences of the primers to be used in the experiment. Total RNA was extracted from the blood samples as described above and the cDNA was synthesized by applying PrimeScript^TM^ RT reagent Kit with gDNA Eraser (Takara Bio, Japan). For quantification, TransStart Top Green qPCR supper Mix (TransGen Bio, China) was used to conduct real-time PCR using the ABI PRISM 7500 Sequence Detection System. All PCR assays followed the protocol: 5 min at 42°C and 10 s at 95°C, followed by 40 cycles at 95°C for 5 s and 60°C for 30 s. Both target and reference (GAPDH) genes were amplified in triplicate per sample. The comparative threshold cycle (2^−ΔΔCT^) method was used to calculate the gene expression.

**TABLE 1:** RT-qPCR primers.

Compare groups	Gene	Primer sequence (5′-3′)
RSV-F vs. AL	P21	Forward: CTGGTGATGTCCGACCTGTT
		Reverse: CAGGGCAGAGGAAGTACTGG
	Irf9	Forward: GAGCAGGTGGAGTTTCCCAA
		Reverse: GCAAAGGCGCTGAACAAAGA
RSV-F vs. AS02	Irf7	Forward: AGCCCTCTGCTTTCTAGTGATG
		Reverse: TCGTAAACACGGTCTTGCTCC
	Akt2	Forward: TGTGCAAAGAGGGCATCAGT
		Reverse: CTTCTTCAGCAGTCCAGCCA
RSV-F vs. AS03	CD21	Forward: GCCCCGATCCAGAAGTCAAA
		Reverse: TCCAGGGGGAAATCGAGCTA
	Gsk3a	Forward: CGCCAACCAGGGAACAAATC
		Reverse: GGATGGACAGTTCACCAGGA

### Statistical Analysis

The difference between groups was compared using one-way or two-way ANOVA where appropriate. *P* < 0.05 was considered statistically significant. Statistical analysis were performed using GraphPad Prism 5 software.

## Results

### Antibody Response

**FIGURE 2:**
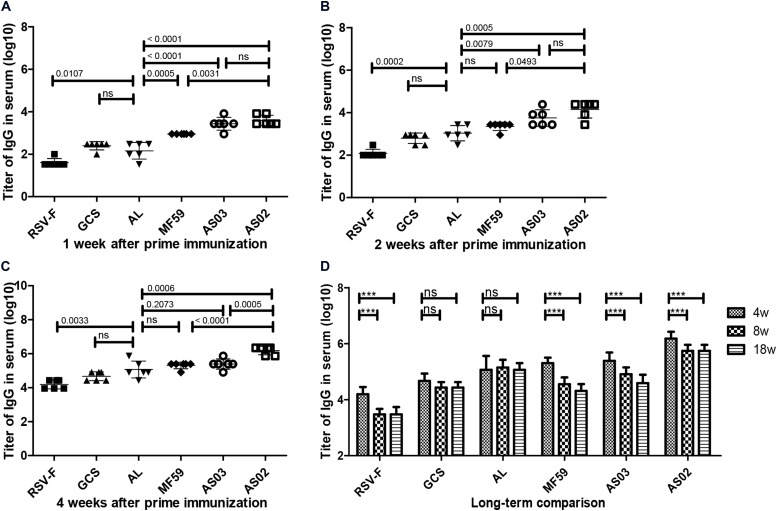
RSV-F-specific IgG levels in the sera of mice intramuscularly immunized with various adjuvanted vaccines and RSV-F alone at weeks 0 and 2. The sera of six mice in each group were separately detected at 1 **(A)**, 2 **(B)**, 4 **(C)**, 4, 8 and 18 **(D)** weeks by ELISA. Results are shown as mean ± SD of antibody titers calculated from all six mice in each group. “ns” *P* ≥ 0.05, ***P < 0.001 between two different groups.

**FIGURE 3:**
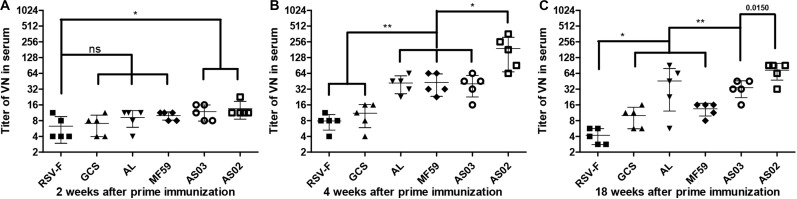
RSV/A/long-neutralizing antibody levels in the sera of mice intramuscularly immunized with various adjuvanted vaccines and RSV-F alone at weeks 0 and 2. The sera of five mice in each group was separately tested at 2 **(A)**, 4 **(B)**, and 18 **(C)** weeks by plaque assay. Results are shown as mean ± SD of neutralizing antibody titers calculated from all five mice in each group. “ns” *P* ≥ 0.05, *P < 0.05, **P < 0.01 between two different groups.

To investigate the effect of various adjuvants on immune responses, pre-fusion RSV-F-specific IgG levels were determined for up to 18 weeks (Figure 2). One week after the first vaccination, all adjuvant groups showed significantly higher antibody titers than RSV-F alone (Figure 2A). GCS induced a comparable antibody level to Alhydrogel, while squalene-based emulsion adjuvants showed higher antibody titers than GCS and Alhydrogel. Among squalene-based emulsion adjuvants, AS02 and AS03 displayed superior performance to MF59 in antigen-specific antibody levels. Two weeks after the first vaccination, each group showed increased antibody titers (Figure 2B). However, all groups followed the same trend: AS02 > AS03 > MF59 > GCS and Alhydrogel (ranked in order of adjuvant activity). At week 2 after the boost, antibody levels in all groups were 100 times higher than week 2 (Figure 2C). The order of adjuvant activity was unchanged, except that AS02 showed a significant higher antibody titer than AS03. To illustrate the potential of adjuvant-induced persistent antibody responses, antibody levels in all groups at weeks 8 and 18 after the first vaccination were also determined and compared with week 4 (Figure 2D). Interestingly, GCS and Alhydrogel showed a sustained antibody response, while other groups showed gradually decreasing antibodies. However, it should be noted that at all time points, AS02 exhibited higher antibody titers than the other adjuvants. Neutralizing antibody assays also showed similar trends to antigen-specific antibody levels (Figure 3). These results indicate that squalene-based emulsion adjuvants are efficient in stimulating antibody production, and AS02 containing MPL and QS21 performed better than other adjuvants in both antibody production and sustained antibody response.

### Antibody Subtypes and Cytokine Assay

**FIGURE 4:**
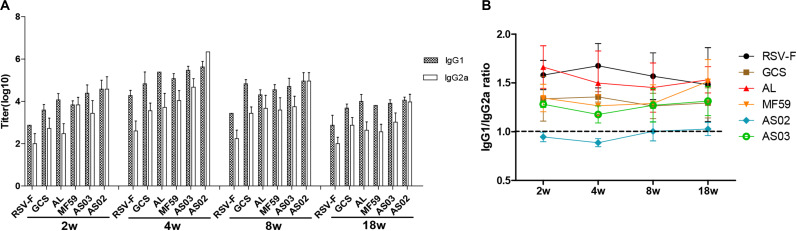
RSV-F-specific IgG1 and IgG2a titers **(A)** and IgG1/IgG2a ratios **(B)** in the sera of mice intramuscularly immunized with various adjuvanted vaccines and RSV-F alone at weeks 0 and 2. The sera of six mice in each group were separately detected at 2, 4, 8, and 18 weeks by ELISA. Results are shown as mean ± SD of antibody titers calculated from all six mice in each group.

To further reveal immune response types induced by various adjuvants, RSV-F specific serum IgG1 and IgG2a levels were determined at weeks 2, 4, 8, and 18 after the first vaccination (Figure 4). As expected, the RSV-F alone group displayed a higher IgG1 titer than IgG2a at all sampling points. Similar immune response types are also found in mice inoculated with GCS, Alhydrogel, MF59, and AS03, but with higher IgG1 and IgG2a levels than RSV-F alone (Figure 4A). These results suggest that GCS, Alhydrogel, MF59, and AS03 can significantly improve both Th1 and Th2 immune responses, but cannot affect the immune response type. In contrast, AS02 elicited strong and comparable IgG1 and IgG2a responses, significantly enhancing Th1 immune response, which is probably due to MPL and QS21. In all groups, IgG1 and IgG2a gradually decreased after week 4. However, no change occurred in immune response type for each group (Figure 4B).

**FIGURE 5:**
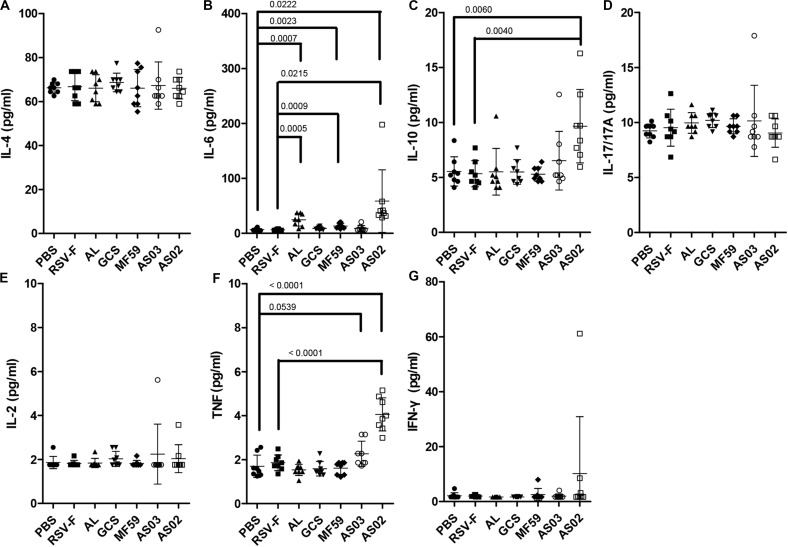
Cytokine levels in the sera of mice intramuscularly immunized with various adjuvanted vaccines and RSV-F alone at weeks 0 and 2. Sera were collected at day 16. IL-4 **(A)**, IL-6 **(B)**, IL-10 **(C)**, IL-17/17A **(D)**, IL-2 **(E)**, TNF **(F)**, and IFN-γ **(G)** were detected by Luminex. Results are shown as mean ± SD of cytokine levels calculated from all eight mice in each group. Groups that are significantly different from PBS group and RSV-F group are marked with *P*-values.

Cytokine responses following vaccination were also compared using serum from mice inoculated with adjuvanted RSV-F vaccines 2 days after the second immunization (Figure 5). AS02 group induced a significant increase in the expression of IL-6, IL-10, and TNF compared to that without adjuvant, while both Alhydrogel and MF59 resulted in higher IL-6 levels than that of RSV-F group. There is no statistically significant difference in the expression levels of IL-4, IL-17/17A, IL-2, or IFN-γ between adjuvanted groups and RSV-F group. IL-6 is known to play a vital role in antibody production and effector T-cell development (43). In a previous report, IL-6 and TNF have been found to contribute a high IgG2a/IgG1 ratio and high expression level of IFN-γ in BALB/c mice when used as molecular adjuvants in a DNA vaccine, which partly agree with that of AS02-adjuvanted group in our study (44). Interestingly, IL-10, a product of Th2 cells that inhibits cytokine synthesis in Th1 cells (45), also showed higher level in AS02-adjuvanted group than other groups. This may, at least partly, suggested AS02’s potential to promote both Th2 and Th1 immune response. Since the effect of cytokines on immune response is dynamic and multifaceted, further study is needed to illustrate the time course of cytokines and corresponding T cell differentiation after vaccination.

### Challenge Test

**FIGURE 6:**
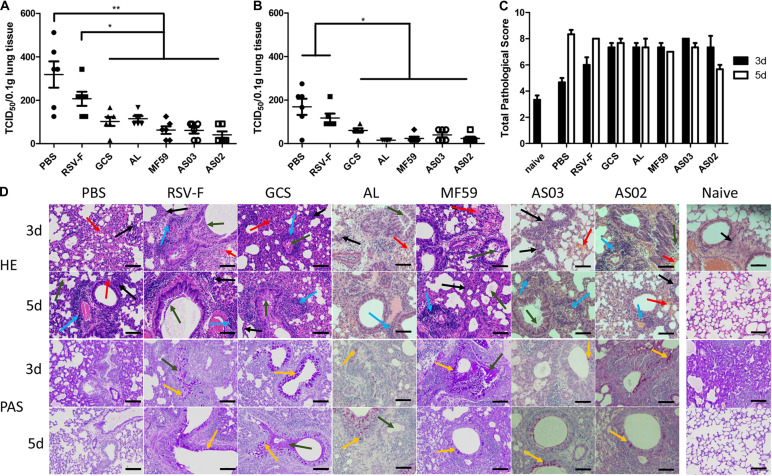
Pulmonary viral loads in lung tissue of immunized mice at day 3 **(A)** and 5 **(B)** after intranasal challenge with RSV/A/long virus. Results are shown as mean ± SD of viral loads calculated from all six mice in each group. Units: TCID_50_/0.1g lung tissue. *P < 0.05, **P < 0.01 between two different groups. Pulmonary pathology scores were calculated according to criteria listed in section “Histopathology” **(C)**. Naive group pathology images were only collected at day 3. Data is shown as mean ± SD of scores calculated from all six mice in each group. Representative HPE and PAS stained mouse lungs at 20× magnification are shown **(D)**. Lung tissue infiltration of lymphocytes and neutrophils (black arrow), perivascular cuffing formed by inflammatory cell infiltration around the local vessels (blue arrow), local bleeding (red arrow), exudation of eosinophilic serous substance in alveolar cavity (green arrow), and bronchial beaker cell (yellow arrow) are marked. Scale bar (black line): 100 μm.

To determine the protective efficacy of adjuvanted RSV-F vaccines, a challenge experiment was conducted in BALB/c mice 14 days after the second immunization. Lung tissue was removed on days 3 or 5 after challenge and the virus titer was measured. On day 3 after challenge, varying virus titers were detected in each group (Figure 6A). Among them, the PBS group showed the highest viral load, followed by the RSV-F alone group. Other groups inoculated with adjuvanted vaccines displayed significantly lower viral loads compared with to PBS and RSV-F alone and gave the order of average viral load of GCS, Alhydrogel > AS02, AS03, and MF59. A similar viral load trend was also observed on day 5 after challenge, but with lower values than day 3 for each group (Figure 6B). These results agree with the neutralizing antibody assay results.

Lung inflammatory responses were also determined and scored according to the evaluation scheme reported by Weiss et al. (42). Alveolar wall lesions, leukocyte infiltration, increasing mucus, and goblet cells were evaluated (Figures 6C,D). Compared with the naïve group, each RSV-challenged group exhibited variable lesion characteristics at days 3 and 5. PBS and RSV-F alone groups showed low lesions on day 3 and exacerbated symptoms from day 3 to 5. In contrast, mice inoculated with GCS-, Alhydrogel-, MF59-, and AS03-adjuvanted vaccines displayed comparable lung lesion levels between day 3 and 5 (Figure 6C). The degree of local lesions is mainly evidenced by alveolar macrophage infiltration and increasing inflammatory cell numbers around the blood vessels. Interestingly, the lesions in the AS02 group decreased from day 3 to day 5, which is probably due to a strong cellular immune response. Pathological change and viral load were inconsistent in lung tissue for PBS and RSV-F alone groups, where pulmonary pathology lagged behind virus clearance. A similar phenomenon is reported in other studies (46, 47). Further study is needed to check the time course of virus clearance and lung inflammatory response more frequently.

### Differential Gene Expression Profiles in Blood Samples

**FIGURE 7:**
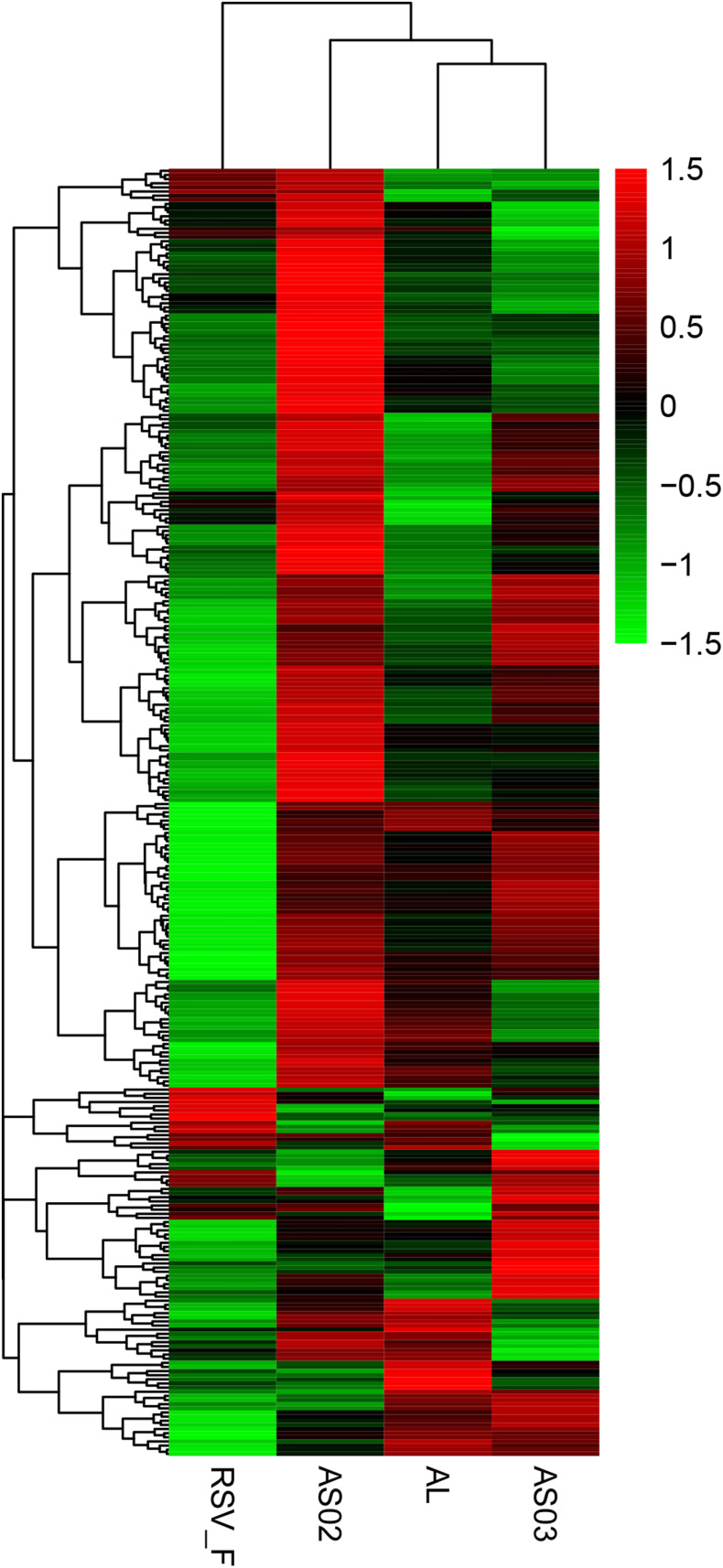
Hierarchically clustered heat maps showing differentially expressed immune-related genes between the adjuvant and adjuvant-free groups. Rows correspond to different genes and columns represent the different samples. The tree above the heat map shows the hierarchical clustering of the samples. The color scale shown in the map illustrate the log10 FPKM. Red and green colors represent high and low expression, respectively. For the description of the genes differentially expressed, please refer to Supplementary Table S1 (Hierarchically clustered heat maps) and Supplementary Table S2 (Immune-related Hierarchically clustered heat maps).

**FIGURE 8:**
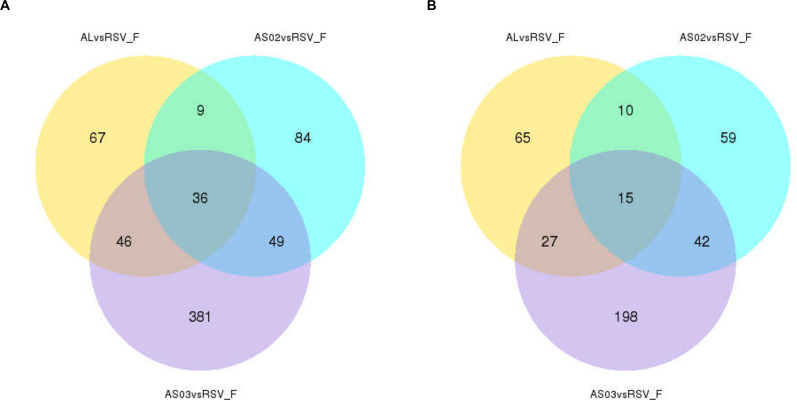
Overlap of immune-related differentially expressed genes (DEGs) among adjuvant groups contrasted with the RSV-F-only group. The number of up- **(A)** and down- **(B)** regulated DEGs in the adjuvant group was shown separately. For the description of the genes differentially expressed, please refer to Supplementary Table S3 (VENN DOWN-regulated) and Supplementary Table S4 (VENN UP-regulated).

To evaluate the gene expression profile of blood samples from immunized mice, the average FPKM of DEGs were hierarchically clustered in the Alhydrogel, AS02, AS03, and RSV-F alone groups. The hierarchical heat map reflects the difference in average immune-related gene expression among the immunized groups (Figure 7). For the description of the genes differentially expressed, please refer to Supplementary Table S1 (Hierarchically clustered heat maps) and Supplementary Table S2 (Immune-related Hierarchically clustered heat maps). To further demonstrate gene expression differences between the adjuvant groups and RSV-F alone group, a VENN map was constructed to express the commonality of up- and down-regulated DEGs (Figures 8A,B). For the description of the genes differentially expressed, please refer to Supplementary Table S3 (VENN DOWN-regulated) and Supplementary Table S4 (VENN UP-regulated). Although there are more DEGs in the AS02 group than the AS03 group (Figure 7), the AS03 group had the most significant DEGs compared to the RSV-F group (Figures 8A,B), which may be related to intra-group differences.

**FIGURE 9:**
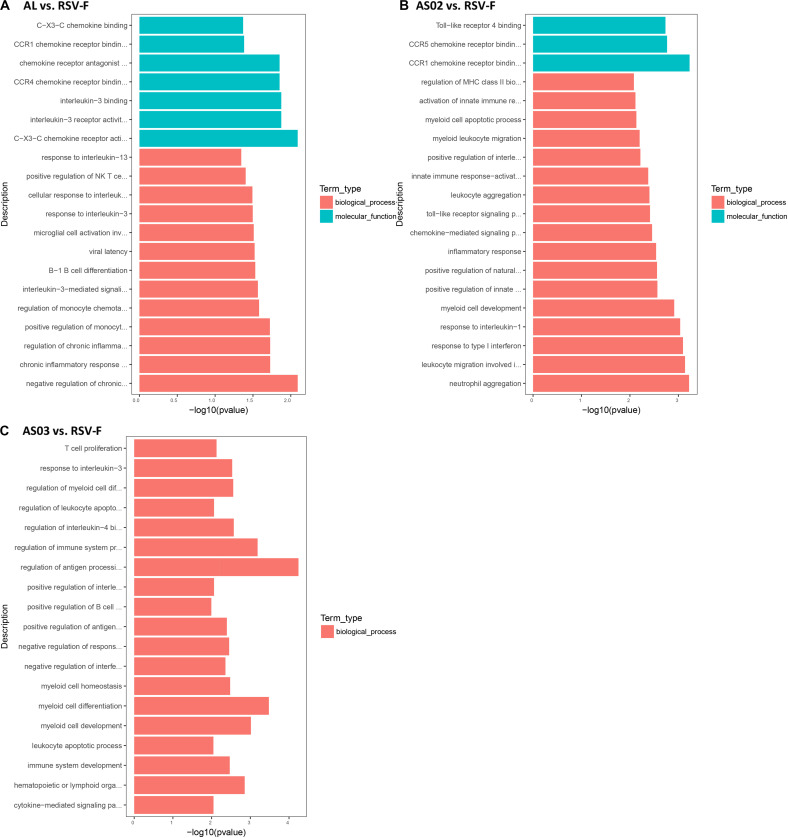
Top 20 Immune-related GO enrichment results. DEGs **(A)** Al vs. RSV-F, **(B)** AS02 vs. RSV-F, and **(C)** AS03 vs. RSV-F were used for GO analysis. The *x*-axis indicates −log (*P*-value). A greater *x*-value represents a more significant difference between the two groups. The *y*-axis represents the function descriptions of the GO terms.

**FIGURE 10:**
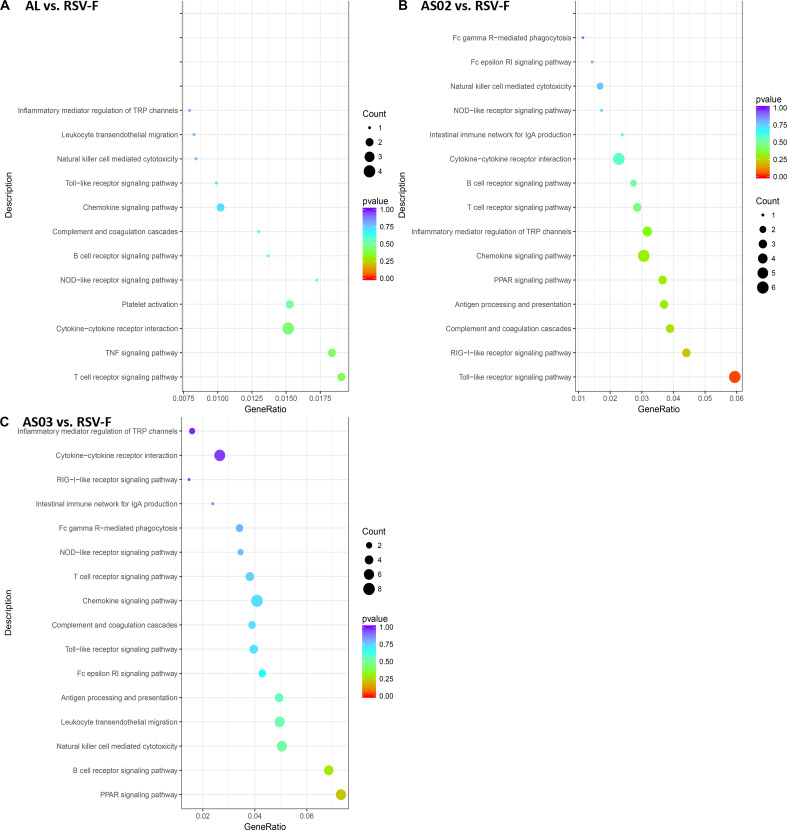
DEG KEGG pathway enrichment after vaccination. DEGs **(A)** Al vs. RSV-F, **(B)** AS02 vs. RSV-F, and **(C)** AS03 vs. RSV-F were used for KEGG pathway analysis using the DAVID functional enrichment database. The *x*-axis is GeneRatio (The ratio of KEGG pathway-annotated genes to the total DEG number). The *y*-axis shows the KEGG pathway. Point size represents the number of DEGs associated with each term. Color reflects the enriched pathway significance level.

The DEGs in each adjuvant group were functionally enriched. The 20 highest activated immune-related GO enrichment results are shown in Figure 9. A brief summary of the GO enriched terms shows that the Alhydrogel group is more similar to the RSV-F group than the other groups. The differential enrichments are centered on Th2-immune-related functions, such as response to CCR4, IL-3, and B1-B cell differentiation. The AS02 and AS03 groups showed a strong influence on the physiology of bone marrow cells. The AS02 group showed significant intervention for the Toll-like receptor, particularly TLR-4, the NF-κB signaling pathway (GO Enrichment “I-κB kinase/NF-κB signaling,” *P*-value 0.028, data not shown) and positive regulation of the biosynthesis process interleukin-2. The differences between the AS03 and RSV-F groups were mainly reflected in the correlation between IL-3 and IL-4. The transcriptome analysis and the cytokine detection results 14 days after the second immunization confirmed that AS02 regulates immune balance by activating TLR-4 and promotes Th1-type immune responses (GO Enrichment “CCR5 chemokine receptor binding,” Figure 9). To find immune-related signaling pathways differentially affected by adjuvant addition, DEGs were again enriched by KEGG pathway analysis (Figures 10A–C). Enriched signaling pathways had low significance, except for the Toll-like receptor signaling pathway in the AS02 vs. RSV-F group, which may be related to fewer detected DEGs caused by intra-group differences. More signaling-pathway-related information may be obtained by adding parallel samples.

**FIGURE 11:**
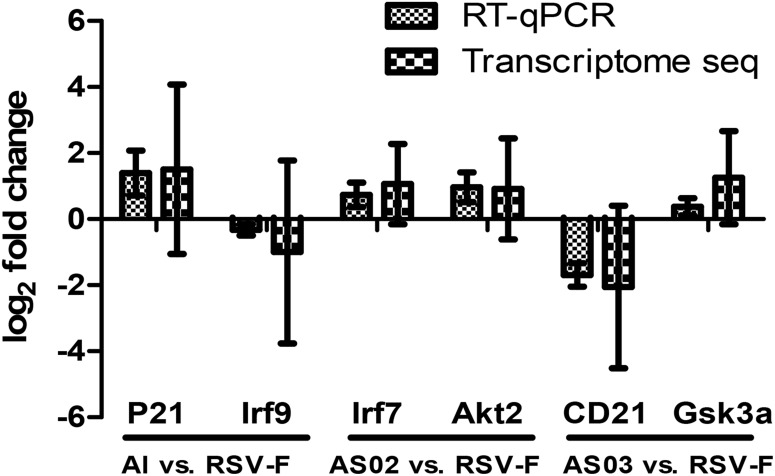
Comparison of transcriptome sequencing and RT-qPCR results of the selected DEGs. Two DEGs from each paired group were selected to confirm the results of transcriptome sequencing. RT-qPCR results used the comparative threshold cycle (2^–ΔΔCT^) method to calculate the gene expression. The *y*-axis shows the ratio of the gene expression between the adjuvant group and the simple antigen group.

To validate the DEGs result in transcriptome sequencing, six genes from three paired groups were selected for RT-qPCR analysis. As Figure 11 shows, the results of RT-qPCR were positively correlated to those of the transcriptome sequencing data, suggesting that the transcriptome sequencing was reliable.

## Discussion

The lack of effective neutralizing antibodies and vaccine-mediated ERD are the main barriers to developing RSV vaccines. Formalin-inactivated RSV-F protein vaccines cause vaccine-mediated ERD and mainly display post-fusion protein conformations (12). The RSV-F protein with pre-fusion conformation is about 80 times more effective than the post-fusion conformation in producing neutralizing antibodies (48). For these reasons, pre-fusion conformation RSV-F was selected as the candidate antigen in this study. The RSV-F antigen was prepared using a recombinant RSV-F subunit vaccine eukaryotic expression system which ensures that the expressed RSV-F has a pre-fusion trimeric structure (13). The capacity of the RSV-F candidate to induce neutralizing antibody was confirmed in a previous study (13). We observed a similar trend between RSV/A/long virus-neutralizing antibody levels and specific antibody titers, which suggested that at least part of the RSV-F protein-induced specific antibody can effectively neutralize the RSV virus (Figures 2, 3).

Although candidate RSV-F antigen can produce neutralizing antibody, low immunogenicity remains a problem, as evidenced by the low RSV-F-specific and neutralizing antibody levels in the RSV-F alone group (Figures 2, 3). For this reason, pre-fusion RSV-F protein was formulated with Alhydrogel, MF59, AS03, AS02, and GCS adjuvants. Among them, AS02 performed better than other adjuvants, producing RSV-F-specific and RSV/A/long-neutralizing antibody at all time points. Although the GCS and Alhydrogel groups showed excellent performance in long-standing antibody production after week 4, antibodies in the AS02 group remained at the highest level until week 18. Besides, the AS02 group also showed excellent viral clearance for RSV/A/long. For example, 3 days after challenge, the AS02 group lung viral load was less than 10^2^ TCID_50_/0.1g lung tissue, which was significantly lower than the PBS group (Figure 6). More importantly, AS02 significantly enhanced Th1 type immune response in immunized mice, which was confirmed by IgG1/IgG2a ratio and cytokine assays (Figures 4, 5). The change in immune response type may prevent ERD, as suggested by the low virus load and fast recovery from viral attack (Figure 6). The clinical manifestation of RSV-caused ERD is a Th2-biased CD4 T cell response, characterized by eosinophils in the peribronchiolar infiltrates and neutrophilic alveolitis in lung histopathology (49, 50). The beneficial change may be due to MPL and QS-21, because AS03 did not affect the original immune response type (Figure 4). MPL is a TLR-4 agonist, while QS-21 promotes APC antigen presentation (27). The DEG KEGG enrichment results indicated that AS02 significantly upregulated Toll-like receptor-related signaling pathways. As previously mentioned, lower antibody affinity is one cause of vaccine-induced ERD. The lack of antibody affinity maturation follows poor Toll-like receptor stimulation (51). AS02 mainly affects the TLR-4 receptor and promotes innate immunity by enhancing “response to interleukin-1,” “positive regulation of innate immune response,” “positive regulation of natural killer cell chemotaxis,” “innate immune response-activating signal transduction,” and “activation of innate immune response” functions (Figure 9). MPL in AS02 may also activate the NF-κB signaling pathway (GO Enrichment “I-κB kinase/NF-κB signaling,” *P*-value 0.028, data not shown) through TLR-4 receptors on immune cells, such as macrophages or dendritic cells at the intramuscular injection site (52). Cytokines such as interleukin-1 can promote monocyte and immature DC recruitment to the injection-site, and mobilize these monocytes into APCs (53–55).

In this study, the Alhydrogel group performed well in neutralizing antibody titer and challenge protection. In addition, the Alhydrogel group has good sustainability in long-term experiments. However, antibody subtypes and GO enrichment results indicated that Alhydrogel-mediated immunity is more inclined to Th2 than other adjuvants (Figures 4, 5, 9), which agrees with previous reports (56, 57). The Th2-biased immune response might adversely affect RSV vaccine safety in children. Previous studies showed that Th2-biased immunity is associated with pulmonary eosinophilia and RSV vaccine-enhanced disease (58–60). MF59 and AS03 were superior to traditional Alhydrogel in antibody production rate, with expressed higher antibody levels at 1 and 2 weeks after initial immunization (Figures 2A,B). This may be due to their ability to recruit immune cells and promote antigen presentation (23, 57, 61, 62). However, both MF59 and AS03 did not alter the original immunological bias of the antigen (Figure 4). In addition, MF59 and AS03 showed significantly decreased antigen-specific antibodies and neutralizing antibody titers after week 4 (Figures 2D, 3C). AS03 showed better long-term serum antibody levels than MF59 (Figures 2D, 3C), which may be associated with α-tocopherol, which promotes antigen transport to lymph nodes (63). The antibody level of the GCS group was low, which may be related to decreased immune cell ability to recognize and capture antigens due to lacking particle carrier properties (64). Further study is needed to illustrate its adjuvant potential as particles or in combination with other adjuvants.

## Conclusion

In this study, RSV-F subunit vaccines formulated with Alhydrogel, MF59, AS03, AS02, and GCS were systematically investigated and compared in terms of antibody production and protection efficacy. AS02 elicited higher and longer-standing antigen-specific antibody and neutralizing antibody responses, followed by squalene-based emulsion adjuvants (AS03 and MF59), Alhydrogel and GCS. Moreover, AS02 significantly improved Th1 type immune response in vaccinated mice and led to a lower lung virus load and faster recovery from viral challenge than other adjuvants, which may be helpful to prevent ERD. The change of immune response is likely caused by the addition of MPL and QS21, because AS03 did not change the original immune tendency of the antigen. Further investigation on gene expression profiles of blood samples also confirmed upregulated Toll-like receptor-related signaling pathways. In comparison with squalene-based emulsion adjuvants, both aluminum and GCS adjuvants showed better durability in immune response, but a slower virus removal after challenge. These findings confirm that AS02 may be a suitable adjuvant for the development of RSV-F subunit vaccines with improved immunogenicity and desired immune response type. Further investigation is needed to illustrate the role of T cell transformation in lung pathology and inflammation process after RSV challenge following vaccination. In addition, the effect of immune routes on the efficacy of adjuvanted RSV F vaccine is under study.

## Data Availability Statement

The datasets generated for this study can be found in the GEO, https://www.ncbi.nlm.nih.gov/geo/query/acc.cgi?acc=GSE143617.

## Ethics Statement

The animal study was reviewed and approved by the Animal Welfare and Research Ethics Committee at Jilin University.

## Author Contributions

YZa, CJ, and WK contributed to conception and design of the study and provided financial support to the project. JL, DL, YS, and YC were responsible for antigen production and purification. KZ and YLu provided the method to virus challenge. YZe, LB, HZ, and YL performed the experiments and organized the database. YZe performed the statistical analysis and wrote the first draft of the manuscript. YZa reviewed and revised the manuscript. All authors contributed to manuscript revision, read and approved the submitted version.

## Conflict of Interest

JL, DL, and YS were employed by Changchun BCHT Biotechnology Company. The remaining authors declare that the research was conducted in the absence of any commercial or financial relationships that could be construed as a potential conflict of interest.

## Footnotes

1 http://www.genome.jp/kegg/

## Supplementary Material

TABLE S1 Hierarchically clustered heat maps.

TABLE S2 Immune-related Hierarchically clustered heat maps.

TABLE S3 VENN DOWN-regulated genes.

TABLE S4 VENN UP-regulated genes.

DATA SHEET S1 Supplementary material for RSV-F protein information.
