# Supplementary material for: Respiratory Syncytial Virus F Subunit Vaccine With AS02 Adjuvant Elicits Balanced, Robust Humoral and Cellular Immunity in BALB/c Mice
Source: Front Immunol. 2020 Sep 11;11:526965. doi: 10.3389/fimmu.2020.526965 (PMC7516270; doi:10.3389/fimmu.2020.526965)
Supplement: DATA SHEET S1 — Supplementary material for RSV-F protein information. [file Data_Sheet_1.PDF]

Supplementary Material

1.1 Supplementary Figures

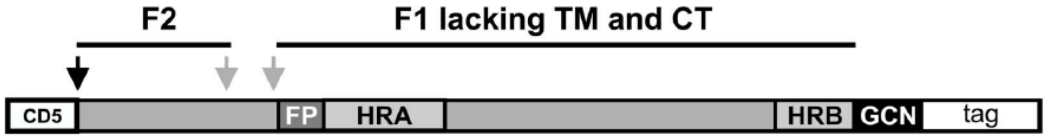

Figure S1. Schematic representation of recombinant soluble RSV F protein construct. RSV F protein without transmembrane domain and cytoplasmic tail were genetically fused to a CD5 signal peptide (CD5) and to a GCN4 trimerization motif (GCN) followed by a His-terminal tag (tag). The tag contains a LysM peptidoglycan binding domain. The F2 and F1 subunits of F are indicated. Black arrow indicates protease cleavage site. Furin cleavage sites are indicated by grey arrows. The approximate location of the fusion peptide (FP), heptad repeat A (HRA) and B (HRB) is also shown. For more detailed information, please refer to previously published reports [1, 2].

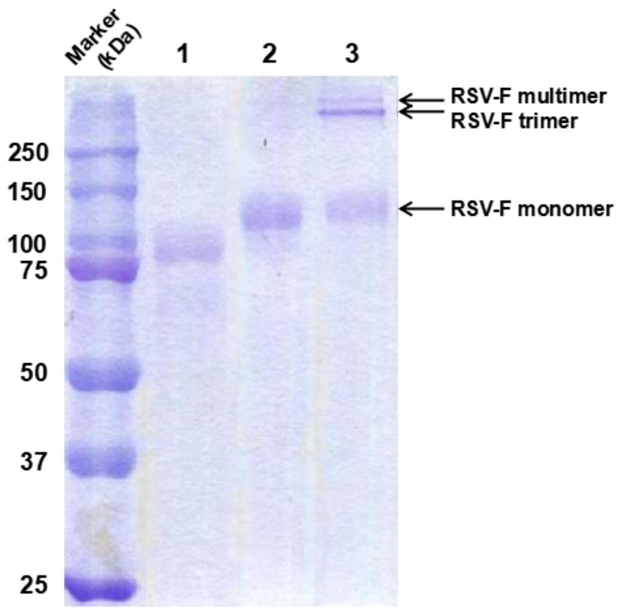

Figure S2. SDS－PAGE analysis of purified recombinant pre-fusion RSV-F subunit antigen. The samples were processed differently before subjected to electrophoresis and were detected using direct staining with Coomassie Brilliant Blue. Marker, Precision Plus Protein^{TM} Standards Dual Color (BIO-RAD) in kD. Sample 1, reduced by mercaptoethanol and heated at 96℃; Sample 2, heated at 96℃ without reduction; Sample 3, without reduction and heating. The results of SDS-PAGE showed that the purified RSV-F antigen was mainly in the form of trimers, and contained a small amount of multimers and monomers. In the process of sample processing, the addition of reducing agent and heating may cause the disintegration of the trimer.

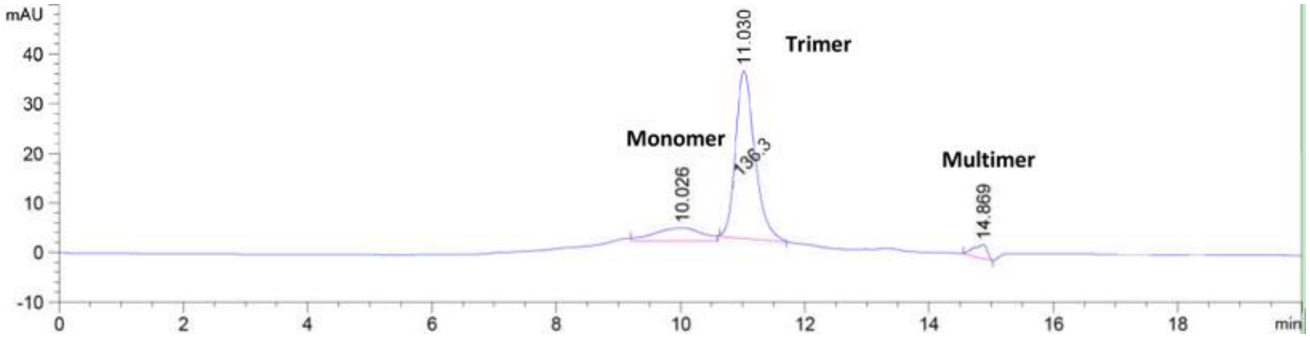

Figure S3. HPLC analysis of purified recombinant pre-fusion RSV-F subunit antigen. 25 µl purified antigen is injected. Chromatographic condition is as follows. Column: Agilent Bio-SEC-5, 300SB-C8, 4.6*300 mm, 5 micron; Mobile phase: 0.1 M PB + 0.3 M Na_2SO_4 (pH 6.8); Flow rate: 0.35 ml/min; Pressure: 40 bar; Temperature: 25 ℃; Wavelength: 280 nm. The result shows that the trimer occupies a large proportion in the purified antigen.

1.2 Supplementary files

Two excel files, named as Table 1 (Hierarchically-clustered heat maps) and Table 2 (Immune-related Hierarchically-clustered heat maps), have been separately added in supplementary materials to support Figure 7 Hierarchically-clustered heat maps

Two excel files, named as Table 3 (VENN DOWN-regulated) and Table 4 (VENN UP-regulated), have been separately added in supplementary materials to support Figure 8 Overlap of immune-related DEGs.

Reference

1. Rigter A, Widjaja I, Versantvoort H, Coenjaerts FE, van Roosmalen M, Leenhouts K, Rottier PJ, Haijema BJ, de Haan CA: **A protective and safe intranasal RSV vaccine based on a recombinant prefusion-like form of the F protein bound to bacterium-like particles.** *PLoS One* 2013, **8:**e71072.
2. Widjaja I, Rigter A, Jacobino S, van Kuppeveld FJ, Leenhouts K, Palomo C, Melero JA, Leusen JH, Haijema BJ, Rottier PJ, de Haan CA: **Recombinant Soluble Respiratory Syncytial Virus F Protein That Lacks Heptad Repeat B, Contains a GCN4 Trimerization Motif and Is Not Cleaved Displays Prefusion-Like Characteristics.** *PLoS One* 2015, **10:**e0130829.
